# Sexual and reproductive health while living with rheumatoid arthritis: The impact of the disease stage on patient perspectives

**DOI:** 10.1371/journal.pone.0302284

**Published:** 2024-04-26

**Authors:** Loraine Ledón-Llanes, Irazú Contreras-Yáñez, Guillermo Arturo Guaracha-Basáñez, Salvador Saúl Valverde-Hernández, Maximiliano Cuevas-Montoya, Ana Belén Ortiz-Haro, Virginia Pascual-Ramos

**Affiliations:** 1 Department of Biology of Reproduction, Instituto Nacional de Ciencias Médicas y Nutrición Salvador Zubirán (INCMyNSZ), Mexico City, Mexico; 2 Department of Immunology and Rheumatology, INCMyNSZ, Mexico City, Mexico; Indiana University School of Medicine, UNITED STATES

## Abstract

**Background:**

Rheumatoid arthritis (RA) is one of the most prevalent rheumatic diseases that harms all aspects of patients’ lives, including sexual and reproductive health (SRH), often neglected in patients’ care. The study aimed to explore the sexual and reproductive experiences of Mexican outpatients with RA from a narrative perspective.

**Patients and methods:**

From July 2020 to October 2021, 30 adult patients with RA from the Department of Immunology and Rheumatology outpatient clinic of a national referral center for rheumatic diseases had in-depth interviews audiotaped, transcribed, and analyzed using a thematic analysis approach. Results are presented in a descriptive and interpretative manner and integrated into a theoretical model for the topic understanding.

**Results:**

Five intertwined major themes emerged: I) RA onset: Absence of SRH contents, II) Healthcare for RA: Emerging SRH contents, III) RA’s impact: Proliferation of SRH contents, IV) Coping with the process of living with RA: SRH-related strategies, and V) The impact of the COVID-19 pandemic on patients’ experiences: Increased SRH burden. SRH contents emerged through these major themes (but at RA onset), mostly when inquired and mainly when narrating the RA impact and coping. Patients identified that RA affected their couple dynamics, sexual function, and reproductive project. The SRH care was considered relevant but limited and focused on reproductive contents. It worsened during the COVID-19 pandemic. We proposed a theoretical model where patients’ SRH experiences are embedded across their RA biography and integrated with the RA impact and the copy with the disease process. These intertwined experiences were also evident during the COVID-19 pandemic, which challenged participants’ biopsychosocial resources.

**Conclusions:**

The sexual and reproductive experiences narrated by the RA outpatients concerning their disease-related biography showed that even when the SRH appeared as not prioritized at the disease onset, it was widely expressed during the process of living and coping with the disease and was additionally affected by the COVID-19 pandemic.

## Introduction

Sexual health (SH) is a complex and evolving construct that harbors critical conceptual elements, including its relationship with reproductive health (RH). From a positive, holistic, and human rights approach, sexual and reproductive health (SRH) evokes well-being, sexual pleasure, its relevance throughout the individual’s lifespan, its diverse expression, its condition of being critically influenced by gender norms, roles, expectations, and power dynamics, and the need to be understood within a specific social, economic, political and health contexts [1–3]. In the past decades, the SH concept has evolved, and even today, academic debates persist concerning what it means, includes, and is related to [2]. The same evolution is expected to occur individually in constructing the concept.

Rheumatoid arthritis (RA) is one of the most prevalent chronic inflammatory conditions, with a worldwide representation [4]. The disease has unique characteristics among patients from the Latin-American region, such as a younger age at presentation (almost ten years earlier) and an extremely female preponderance [5,6]. RA influences all aspects of life, including the SRH. Impaired SRH has been attributed to musculoskeletal pain, high fatigue levels, mental distress, functional limitations, low self-efficacy, and gender issues [7]. One of the SRH dimensions, sexual function, is relevant across health and disease stages [8] and can be impaired in patients with RA. The disease has been identified as a risk factor for sexual dysfunction [9], which involves physical problems, impaired marital emotions and relationships, pain, fatigue, functional weakness, anxiety, negative self-image, reduced sexual desire, hormonal imbalance, and drug side effects [10]. Meanwhile, reduced fertility affects women and men with RA and has been related to disease activity, pharmacological interventions, impaired sexual function, and personal choices [7,11].

SRH is often neglected in the care and management of patients with rheumatic diseases due to several barriers, including insufficient patient and physician skills, lack of training of healthcare providers, and lack of patient access to specialized services [7]. Our group has performed three previous studies on women and men living with RA [12–14]. The first study revealed that Mexican patients with RA perceived their SRH as a critical component of their general health and wished to address the topic with healthcare professionals [12]. The second study interpreted SH and RH definitions of the same sample of patients. We observed that SH contents were defined by related diseases, actions directed to prevention, and mentions of the couple; meanwhile, RH tended to be defined by a primarily biological perspective of the reproductive function [13]. The third study evidenced that most female patients had impaired sexual function, while it was present in a few male patients. Male gender, fatigue, and cohabitation with the couple were protective against impaired sexual function [14].

The COVID-19 pandemic context should be added to previous reports for a more profound understanding. There is accumulating evidence that the COVID-19 pandemic might result in additional collateral damage for patients with chronic conditions due to issues in medication supply and economic setbacks to society [15]. These factors have led to a humanitarian crisis in Latin America, where public life is characterized by fragile health systems and long-standing and pervasive inequity [16].

With the above considerations in mind and to better comprehend patients’ SRH perspectives, the present study explored the sexual and reproductive experiences of a group of Mexican outpatients with RA while living with the disease, including throughout the COVID-19 pandemic. To attain this purpose, we based our work on a biopsychosocial theoretical model of adjustment to the RA that involves the stress and coping paradigm incorporating the positive aspects of psychosocial functioning and adjustment [17] through a biographic-narrative methodology [18]. This approach allowed us to contextualize the SRH biography while living with the RA and build an understanding proposal inductively, starting from the participant’s narratives.

## Materials and methods

### Ethics

The Research Ethics Committee of the Instituto Nacional de Ciencias Médicas y Nutrición Salvador Zubirán (INCMyN-SZ) approved the study (reference number: IRE-3388-20-21-1). All the patients included provided written informed consent. Patients had a free space to express themselves. When necessary, they were offered access to health care services. The interviewer had training in properly managing different psycho-affective situations during the in-depth interviews.

### Setting and participants

The INCMyN-SZ is a tertiary care and national referral center for rheumatic diseases in Mexico City. Participants were identified from the Department of Immunology and Rheumatology outpatient clinic (ORCID). Adult RA patients (≥18 years of age) waiting for consultation between July 2020 and October 2021 were invited to participate (all the coauthors participated in the recruitment process). They were selected considering the heterogeneity criteria regarding age, sex, sexual orientation, the age at RA diagnosis, the time living with RA, the disease activity status based on the attending rheumatologist criteria, the state of having (or not) a partner, and children, and the parenthood desire. We considered an initial sample of 15 patients, and we used inductive thematic saturation (the point at which the obtained information became repeated or redundant) [19] aimed to identify new themes and codes [20,21]. The standardized process for attaining saturation considered the arising of the same topic in three consecutive interviews (three investigators: LLL, ICY, GGB). Thirty patients were integrated into the final sample.

All participants’ RA diagnosis was based on the treating rheumatologist’s criteria. Exclusion criteria were RA patients with overlapping rheumatologic syndrome (except Secondary Sjögren Syndrome) and patients with uncontrolled comorbidity requiring treatment intensification or palliative care.

### Interview

Four investigators (LLL, ICY, GGB, VPR) proposed and consensually agreed on the general topics of the in-depth interview guide, which was directed at exploring participants life stories living with RA, particularly their sexual and reproductive experiences from the beginning of the disease to the current time, which was coincident with the COVID-19 Pandemic. The in-depth interview guide included general and open questions to allow the participants to structure their narratives. It also incorporated themes that emerged during patients’ narratives. Interviews had a mean duration of 57 minutes (27 to 96), and one researcher (Ph.D. in Psychology and trained in SH: LLL) conducted all the interviews in a private area at the ORCID.

### Analysis

For the sample description, we carried out a simple quantitative analysis. We present the categorical variables as frequencies and the continuous variables as median and interquartile range.

All in-depth interviews were audiotaped and transcribed with correct spelling and congruent with the specific way each patient expressed her/himself. The transcripts were analyzed using a thematic analysis [22,23], which was done as a recursive and cyclic process. Three investigators (LLL, ICY, GGB) read the transcripts one first time before coding to become familiar with the text’s content and individual ways of expression. They independently coded the transcripts and identified the major themes with their corresponding categories and sub-categories associated with the discursive fragments and quotations in an inductive and iterative process [24,25]. They added new codes as needed, and through systematic meetings, with the participation of a fourth investigator (VPR) in the role of an external judge, they discussed the findings, built consensus regarding the applicability and relevance of themes and categories, and refined them and reduced data, until building the complete codebook and the theme framework. Finally, we built a theoretical model (all the co-authors were involved) supported by the data, and after refining the initial interpretations from different angles and perspectives from a narrative, integral, and evolutionary qualitative approach [22,23]. We followed the standards for Reporting Qualitative Research (SRQR) guidelines (Please refer to the S1 Table. Standards for Reporting Qualitative Research (SRQR)” checklist for qualitative research).

We present the results in a descriptive and interpretative manner. The present report includes the major themes expressed logically and integrates the main ideas from the analysis related to the data (quotations). The final structured themes were organized considering the fulfillment of the internal homogeneity, the external heterogeneity, and the coherence and consistency characteristics [23].

## Results

We are presenting the main results as patients´ characteristics, the major themes description, and an integrated and evolving proposal built upon the patients’ perspective regarding their SRH living with RA.

### Patients’ characteristics

Patients interviewed were primarily middle-aged (40 years of age [34–49]) females (n = 25) and had 12 years of formal education (9–17). Nineteen patients had a formal job, 17 lived together, 19 (61.3%) were in a relationship, and 25 were from urban areas. Patients had substantial disease duration (12 years [7–16]), and 23 patients were in remission (RAPID3 <3). The majority had some comorbidity (n = 28). The more frequent chronic conditions were dyslipidemia (n = 17), hypertension (n = 11), hypothyroidism (n = 5) and diabetes mellitus (n = 4). Also, 17 patients had children, and the number was 2 (1–2). Twenty-nine patients self-referred as cisgender, and 13 referred sexual discomforts. Finally, 15 patients used at least one contraception method.

### Major themes description

Patients were asked to narrate their life stories living with RA, particularly their SRH experiences, from RA onset to when the interview was conducted. Five major themes emerged: I) RA onset: Absence of SRH contents, II) Healthcare for RA: Emerging SRH contents, III) RA’s impact: Proliferation of SRH contents, IV) Coping with the process of living with RA: SRH-related strategies and V) The impact of the COVID-19 pandemic on patients’ experiences: Increased SRH burden.

**Fig 1:**
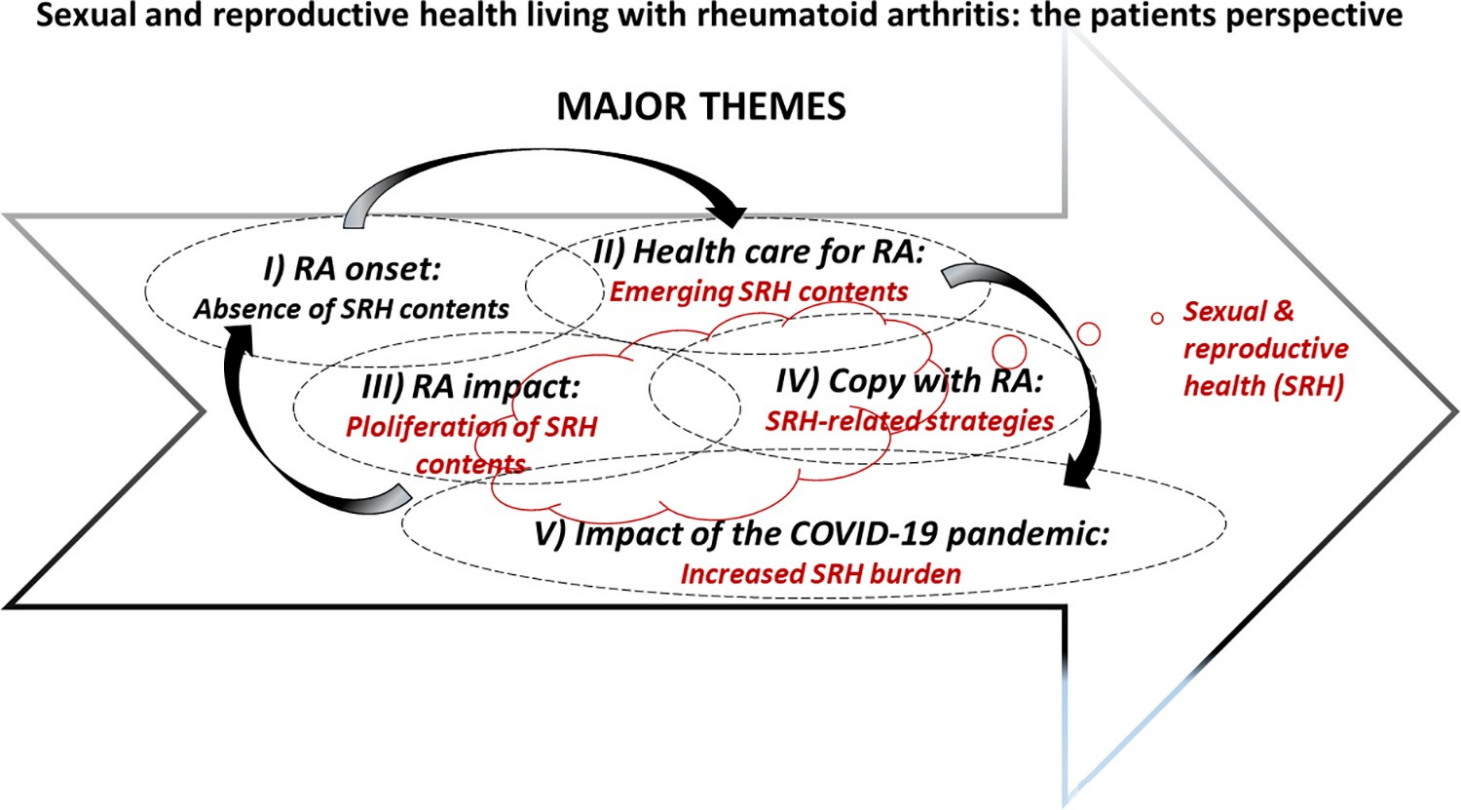
Major themes when inquiring sexual and reproductive health during the process of living with rheumatoid arthritis.

Major themes emerged without a specific order and interrelatedly. The "RA onset: Absence of SRH contents" major theme was linked to "Healthcare for RA: Emerging SRH contents" and "RA’s impact: Proliferation of SRH contents" major theme to "Coping with the process of living with RA: SRH-related strategies." Meanwhile, "The impact of the COVID-19 pandemic on patients’ experiences: Increased SRH burden" major theme emerged when structuring the impact of the disease during the interview (**Fig 1**).

As detailed below, SRH contents appeared in all major themes but for "RA onset: Absence of SRH content." They were persistent, enriched, and came into view spontaneously in the context of the "RA’s impact: Proliferation of SRH contents" and the "Coping with the process of living with RA: SRH-related strategies" major themes. They also appeared in the "Healthcare for RA: Emerging SRH contents" and "The impact of the COVID-19 pandemic on patients’ experiences: Increased SRH burden" major themes primarily from the interviewer’s inquiry.

#### SRH narratives during the RA onset and the first steps in looking for healthcare

None of the participants revealed SRH content related to the RA onset, even when challenged. After inquiring, they appeared for the first time during the narratives related to the “Healthcare for RA: Emerging SRH contents” major theme.

**Fig 2:**
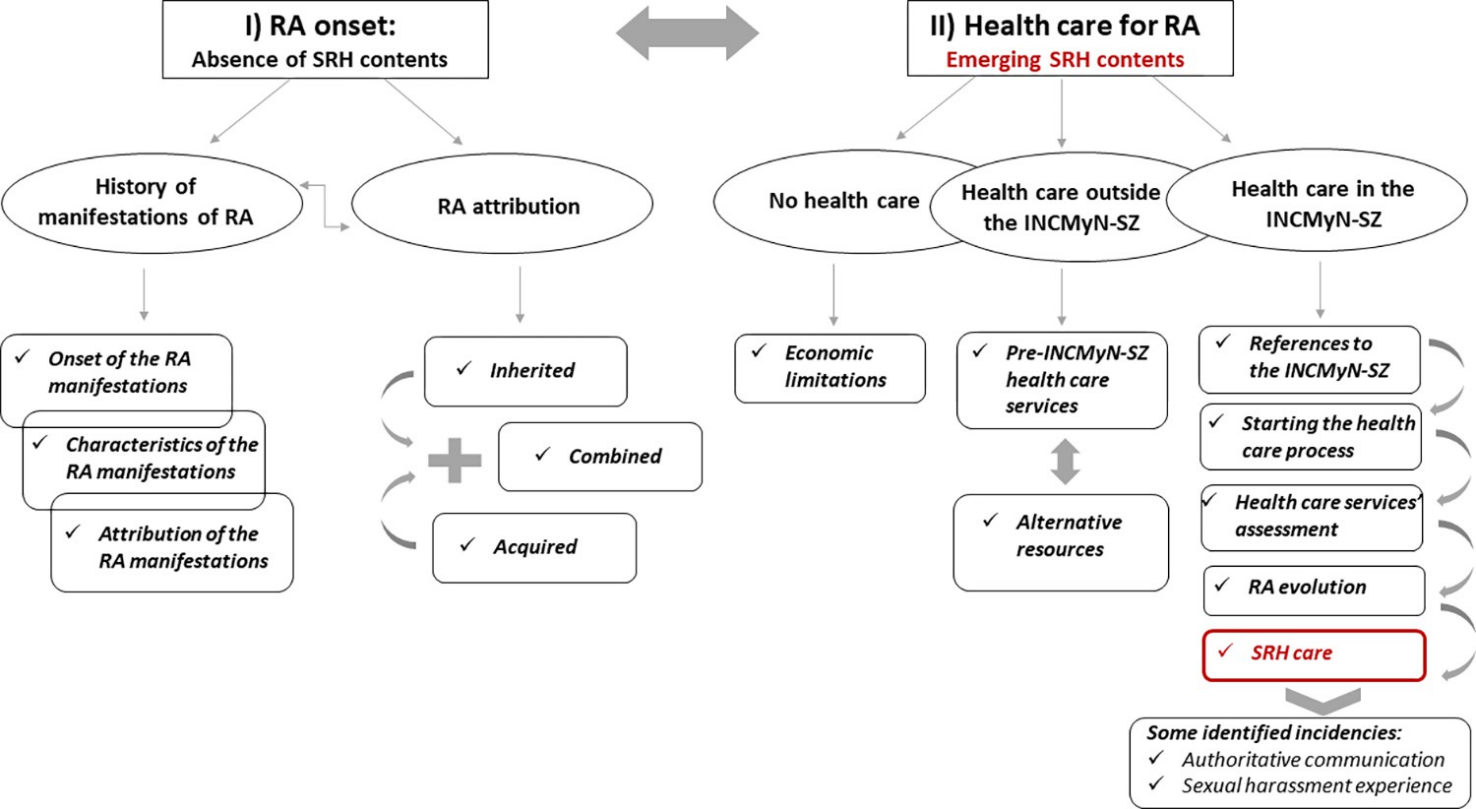
The onset of rheumatoid arthritis and the first steps looking for health care: The scarcity of sexual and reproductive health´s contents.

The narrative of the disease manifestations particularly enriched the RA onset in terms of their initial stages, specific characteristics and attributions, the moments in which they became more intense, and their relationship with other coexistent health conditions. The first manifestations of RA were associated with some life events and activities or other health processes, but for some patients, the beginning of these manifestations was "spontaneous." They appeared mainly in acute presentation, although intermittent and gradual presentations were also mentioned. The richest narratives were expressed about the disease-related manifestations (e.j. pain, fatigue, inflammation, stiffness, impaired motility, and limitations in daily activities). They were structured from a sense of limitation (**Fig 2**).

"*But there were times when I really said*. . .: *No*, *I cannot even get up*. *I am so stiff when I have to get out of bed*, *with my hands to help me*, *to lower one foot and then the other foot*, *and wait there for about half an hour*, *to start to feel*, *to massage me and start to feel*, *and that is how*. . ., *I would get up*. . .*" (*woman, 43 years old*)*.

Patients identified the first RA manifestations in different life stages. For example, during childhood and adolescence, their first youth (20 to 30 years old), or later in life (middle age).

"*I remember a scene like that very much*, *that I have it very much nailed down*. *I ran downstairs and said to my mom*: *Look*, *Mommy*, *I cannot close my hands*. *I thought that was funny; it did not scare me or anything*. *I mean*, *it was like*. . .: *What is happening to me*? *But it was funny*, *wasn’t it*? *At that time*, *I was in the first year of high school*. . . *I was 15 years old*. . .*" (*woman, 29 years old*)*.

"*But then I told him*, *no*, *for this I was*. . . *what*? *47 years old*. . ., *it was 8 years ago*. . ., *46 years old*. *So*, *I said*: *No*, *well…*, *I don’t know how it feels to spend a year*, *another year*, *then I said by myself*: *Well*, *what have the years weighed on me*? *then they talk*: *Could it be the old age giving on me*?*" (*woman, 54 years old*)*.

The attribution of RA permeated the narratives concerning its onset and was considered hereditary, acquired, or combined. The hereditary attribution was related to having relatives with RA, contributing to looking for healthcare more effectively and quickly. For other patients, the attribution was adjudicated to temporary life and health events, including psychological factors and delayed patients´ effective strategies of seeking healthcare (**Fig 2**).

"*I have three aunts with rheumatoid arthritis*. . . *I began to have discomfort in my fingers and joints*, *and I began to pay attention…; it crossed my mind that perhaps it was my aunt’s disease; one of them told me that they would take me to the rheumatologist*.*" (*woman, 28 years old*)*.

"*I started in January 2018 with extreme fatigue and this…*, *difficulty moving as I usually did (…)*. *When it was time to take a bath*, *I had a hard time rubbing my head and tying my tennis shoes; that was when I said*: *Something is weird; I definitely can’t think of anything more transitory*, *and I decided to study myself*.*"* (woman, 41 years old*)*.

The major theme of “Healthcare for RA: Emerging SRH contents” emerged as closely related to the “RA onset: Absence of SRH contents”. It was generally structured after the narratives about the first stages of living with RA symptoms. Once the first manifestations emerged, the descriptions of both major themes intertwined (**Fig 2**).

Patients described three related paths in accessing healthcare for the disease. Generally, they spend a variable amount of time (months or years in some cases) looking for healthcare services to identify and manage their health condition before arriving at the INCMyN-SZ. Those health services were primarily located in clinics or general hospitals, geographically accessible, and were private or public institutions. Orthopedists and internal medicine specialists were involved in patient care. However, alternative medicine was also used for managing physical limitations (e.g., pain, fatigue, inflammation, stiffness).

"*(*. . .*) I called a doctor that I have (*. . .*) since we were younger*, *and he is the one who regularly assisted me (*. . .*)*. *Doctor says to me*: *(*. . .*)*. *Look*, *I will recommend the phone numbers of some of my colleagues to you*. *He says*: *Give me a chance to check if he is a neurologist*, *a rheumatologist*, *or*. . . *And I asked him*: *What*? *and he told me*: *"Yes*, *I think that what you need is a specialist*, *I would not like you to stop taking care of it because it does not seem like your normal condition*, *ehm*, *I am going to leave you these medications*, *as far as that we got the appointment with the rheumatologist*, *right*? *(*woman, 29 years old).

"*I started to feel bad*, *I would go out in the sun*, *warm up and home remedies*. . ., *put on alcohol*, *a herbal bath with rue*, *Santamaria*, *rosemary*, *basil*, *chamomile*, *lots of herbs*…" (woman, 62 years old).

After a time without achieving RA-related symptom control or improvement, or because of severe functional decline due to symptoms flaring, in some cases because RA diagnosis was integrated, or after being informed about the existence of a medical specialty named “Rheumatology” with a reference service located in the INCMyN-SZ, some patients finally arrived at the Institution. Other patients arrived more directly, advised by significant others, relatives, and/or health professionals. Some patients reported economic limitations impeding access to healthcare.

In many cases, the description of patients´ healthcare experiences in the INCMyN-SZ was favorable, primarily considering the effectiveness in attenuating and controlling symptoms and recovering physical function, motility, autonomy, and well-being. Patient-centered care characteristics were also highlighted by the patients, who expressed an integral approach, systematic disease monitoring, disease activity control, and humanistic aspects such as effective communication, trust, and empathy. Nevertheless, two female participants referred to an incident related to authoritative medical communication and a sexual harassment experience by a healthcare provider, which negatively impacted their healthcare process. Still, they were adequately assisted by the healthcare team.

“*I don’t complain about the attention*, *it’s very good*. . ., *the doctor cares about his/her patients*, *she/he tries to find things that could benefit us the most*.*”* (woman, 58 years old).

At the INCMyN-SZ, SRH contents were mentioned by the patients as insufficient, not always offered, and mainly oriented to female reproductive health, notably to family planning, prevention of unplanned pregnancies, medical treatment in the context of pregnancy, adverse outcomes related to unplanned pregnancy and reproductive organs’ screening. One participant considered the topic of sexuality was missing and/or medicalized. Also, some patients with sexual discomforts and/or specific information needs in this area referred to the lack of an adequate orientation, and the physicians normalized their symptoms and perpetuated their sexual discomforts.

*“I would have liked more information about arthritis*, *about the weight*, *and sexual activity*.*”* (woman, 62 years old).

Interestingly, some patients who referred not receiving any SRH care mentioned during their interview having received information, for example, about family planning, the indication about reproductive organs screening, and/or about the potential effects of the disease on the reproductive process.

#### SRH emerged as a relevant area while living with RA

The "RA’s impact: Proliferation of SRH contents" and "Coping with the process of living with RA: SRH-related strategies" major themes were among the richest in SRH content, which flourished from the most profound patients’ reflections and with a solid affective involvement.

**Fig 3:**
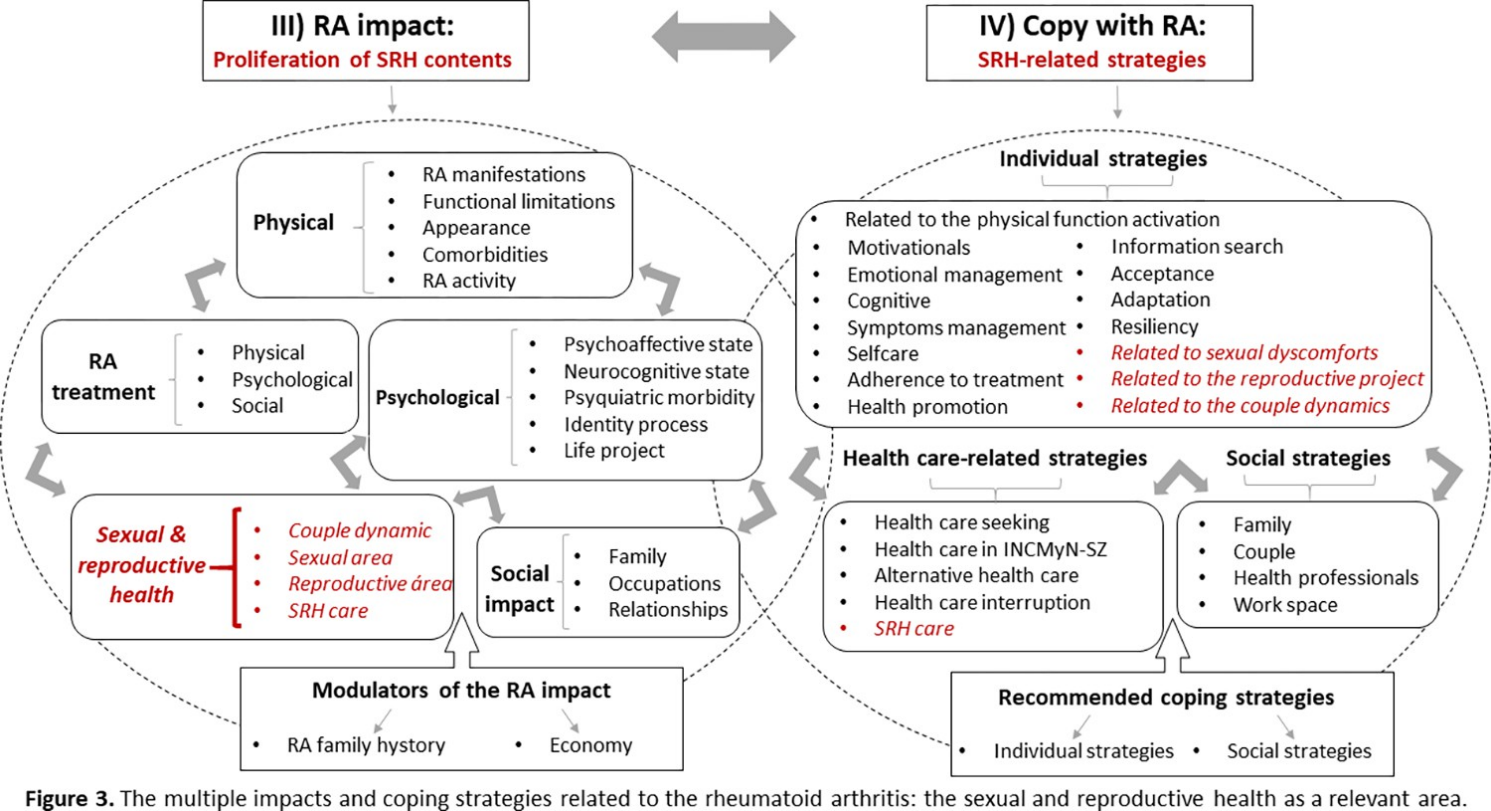
The multiple impacts and coping strategies related to the rheumatoid arthritis: The sexual and reproductive health as a relevant area.

The patients described the disease impact as multiple, severe, and challenging, extending to the physical, psychological, social, and SRH areas. Patients also dedicated a unique space for narrating about the impact of the RA treatment. In parallel, they shared the coping strategies to manage impacts, which were deployed at the individual, social, and healthcare levels and in a linked way (Fig 3).

The RA’s physical impact included disease manifestations, related functional limitations, RA effects on patients’ physical appearance, and disease-associated comorbidities. The psychological impact extended to RA effects on the psycho-affective and neurocognitive status, psychiatric comorbidity, and other psychological processes such as identity and life projects. The social effects of the RA extended to the family and the couple’s relationship but also to additional relationships (friends, co-workers, people in general). The effects of the disease-related treatment also affected the physical, psychological, and social contexts from both an affirmative and an adverse perspective.

“*I began to see the complexity of the disease; for me*, *it has been super strong; I am very afraid that it will cause damage*, *that it will leave me disabled*, *blind or have a cardiac arrest*.*”* (woman, 37 years old).

SRH contents frequently appeared when directly inquired about but with deeper emotional involvement and abundant expression of topics related to the RA effects in the couple, sexuality, reproduction, and SRH care. Contents were primarily focused on the deteriorating effects of the disease on sexual function (expressed in sexual discomforts), the adverse mediation of the physical and psychological disease impact on sexual motivation and performance, and couple dynamics. Additional emerging contents were the decisions related to family planning, the use of contraceptives, and the maternity or paternity desire.

"*In the sexual area specifically*, *it has not affected; at the first time*, *it was complicated; when arthritis was detected because of the pain*, *I did not feel like anything*. *Today*, *I lead my life almost normal*.*" (man*, *41 years old)*.

"*Before I had my hip surgery*, *I could not have sex*. *Since they operated on me*, *I started my sexual life*.*" (woman*, *40 years old)*.

"*(Regarding his sexuality) Yes*, *I feel that it has affected*, *because*. . ., *well no*. . ., *(he makes a silence)*, *it hurts me*. . ., *a lot*, *well*, *the body*. . . *For me*, *it is very painful to have the*. . ., *a good relationship*, *(*. . .*) then my current partner looks for a way*, *like*. . ., *like he is a little more understanding*. *Find a way to*. . ., *but even so*, *I mean*, *maybe I am embarrassed to say it*, *but*. . ., *days ago*, *two weeks ago*, *uhm*, *we had a relationship*, *I really stood still*, *I stayed*, *I did not was able to get up*, *that is*, *I stayed*, *he says*: *What’s wrong with you*? *It is just that it cannot be (he speaks*, *laughing)*. *He says*: *How is it possible*? *I tell him*: *I am really not lying to you*.*" (woman*, *43 years old)*.

"*Well*, *mainly*, *my life plan at 18 was that at the maximum of 24–25 years*, *well*, *being just married*, *having plans for a family*, *something like that*. *Well*, *that was mainly my family plan*. *And when they detected this*, *I did say*: *No*, *well*, *what if my partner does not feel comfortable with someone like me*, *my partner disagrees with*. . ., *because I decided not to have children unless I was sure that they did not suffer from this*.*" (man*, *26 years old)*.

When inquired, patients recognized SRH care as essential. However, they referred to it as limited and focused on reproductive contents, such as the effects of the pregnancy on the women’s health, offspring viability, the relevance of planning the pregnancy and on-time communication with the rheumatologist regarding RA treatment, the prevention of unplanned pregnancies by using effective contraception, and the relevance of regular screening of the sexual and reproductive organs.

“*I suffered a lot in my first pregnancy*, *then I decided not to get pregnant anymore*, *and worse*, *when they detected my arthritis*, *I said no*, *not anymore*.*” (*woman, 42 years old).

“*For me*, *the key to success is perseverance*, *exercising*. . ., *following the treatment instructions*.*”* (woman, 66 years old).

Patients used a broad group of coping strategies to manage RA’s challenges. They mentioned several individual strategies to recover from and/or palliate physical limitations, perpetuate motivations for living with RA, manage the psycho-affective state, and understand and cognitively adjust to living with the disease. One strategy aimed to manage primarily biomedical aspects of the disease, such as symptoms, self-care, adherence to treatment, health promotion, and health information seeking. A more complex individual coping strategy patients referred to was disease acceptance, adaptation to living with this systemic and chronic disease, and resiliency as the ability to adapt and overcome the adverse situations accompanying living with RA. Other specific strategies were aimed at facing the complex demands of living with RA in the sexual and reproductive areas. As part of them, patients mentioned strategies for managing sexual discomforts, the reproductive project (previously planned and/or changed because of the RA), and the demands on the couple dynamics.

Patients also referred to social support from family members (parents, sons and daughters, and other relatives), the couple, the health professionals, and even people from the workplace (co-workers, managers) as an interrelated area with the coping strategies.

"My husband supports me a lot, I mean, really. . . he is the one who covers the expenses, or I do with what I earn, no (she speaks laughing) no, it’s like he has been a support not only economically, but also emotionally, in other words, he has accompanied me throughout the process. . .". (woman, 37 years old).

"The doctor sent me to internal medicine, and there, they asked me for a Pap smear, everything it has to do to me, the breasts too." (woman, 58 years old).

Finally, some categories emerged from both major themes to highlight aspects that attenuate the disease impact and coping with the disease’s most successful strategies. Patients mentioned that having relatives with RA and knowing their experiences and a better economy improved the experiences of living with RA. Also, patients recommended individual and social strategies to face the challenges of living with RA, specifically, having a proactive attitude, reinforcing communication with the health professionals and treatment adherence, and sustaining auto-affirmation strategies aimed at self-validation.

#### The impact of living with RA during the COVID-19 pandemic on SRH burden

The in-depth interviews were conducted during the COVID-19 pandemic, and as a result, this major theme inevitably emerged. However, the interviewer also inquired about it in the SRH context.

**Fig 4:**
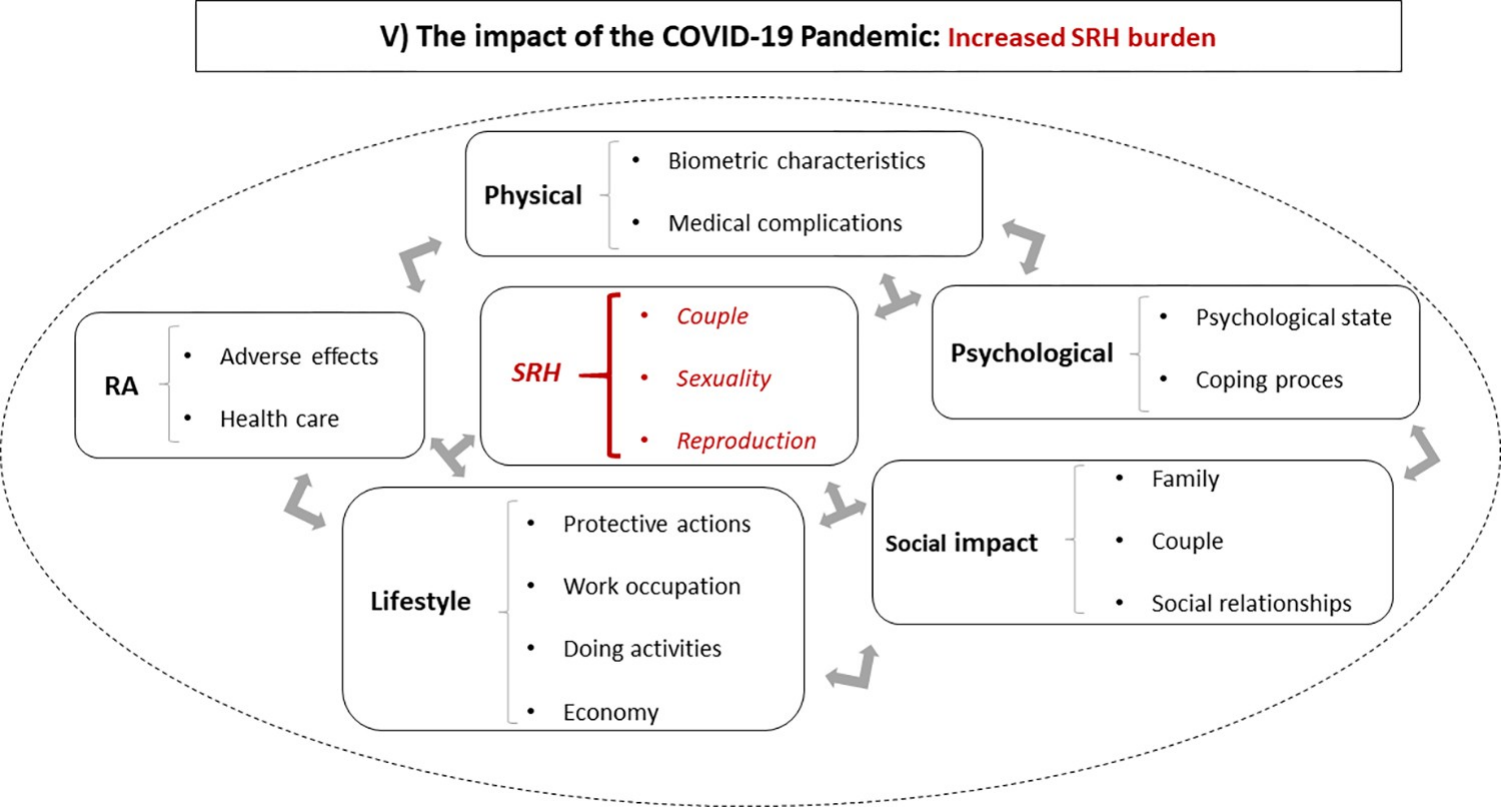
Living with rheumatoid arthritis during the COVID-19 pandemic: Increasing the sexual and reproductive health burden.

Patients’ narratives about the COVID-19 pandemic were related to its impact on biometric characteristics (specifically, body weight) and medical complications resulting from the nasal swab for a SARS-COV2 test (sinus infection, facial palsy). However, patients centered their narratives on the psychological, social, and lifestyle effects of the COVID-19 pandemic (Fig 4). They also identified the deleterious impact of limited healthcare, particularly regarding SRH.

Concerning the psychological area, they mentioned fear, frustration, anger, anxiety, and apprehension related to the possibility of being infected, the vulnerability of living with RA, and the limited access to healthcare during the pandemic. The social effects focused on the family, the couple dynamic, and other social relationships. In the family context, patients mainly referred to topics about contagion, the increasing time spent with the family, the re-structuration of the family dynamics and activities, the emerging conflicts, and its support function. In the couple dynamics, the experiences were centered on the re-organization of the activities, the effects of the physical separation, or the more time in cohabitation. Finally, patients also referred to social distancing and isolation during the COVID-19 pandemic. The perceived psychological and social effects were mediated by lifestyle changes structuring protective actions, such as personal and environmental hygiene and compliance with health authorities’ guides. Other affected areas were work (work interruption, home office modality) and "everyday" activities (domestic, recreational, physical), which had an economic impact on patients’ lives.

"*(*. . .*) well*, *yes*, *my family is more concerned (*. . .*)*. *And the masks*! *And the alcohol*! *(he mumbles) And everything*! *(silence) Well*, *everything that’s going on with the pandemic*, *nothing has happened to me*. . ., *and despite*, *as I am telling you*, *my autoimmune disease*, *even though my system is*. . ., *(silence) let us say my defenses are*. . ., *horribly wrong (he speaks laughing)*, *I am here*, *so*. . . *(silence)*. *I feel*, *yes*, *at first it was kind of stressful*.*" (man*, *35 years old)*.

"*We went out only what was necessary and the use of masks*, *I practically did not take the children out*.*" (woman*, *28 years old)*.

Patients also referred to the COVID-19 pandemic’s impact on the underlying rheumatic disease. The emerging categories evidenced insufficient information about the healthcare process during the pandemic, RA and comorbidities-related treatment changes, shortage of medicines, and RA flares as a result of or during the pandemic.

“*At the time of the COVID-19 pandemic*, *there was a lack of medications*, *and they closed the hospital; I began to feel my discomfort*.*”* (woman, 28 years old).

Finally, focusing on SRH, patients referred to the couple, and the sexual and reproductive functions. They specifically mentioned how the adoption of pandemic-related lifestyles impacted the couple dynamics, how the increased cohabitation in couples could diminish or increase the sexual frequency, and some processes related to the reproductive project decision during the pandemic.

*(Referring to sexuality in couples) “So far*, *we are fine*. *My wife and I wanted to go out to dance a lot*, *and we went out frequently; now I tell you that right now*, *it is not possible; we do not go out*. *Well*, *we liked to go out to eat*, *go to restaurants to listen to live music and dance and eat… (Did you keep doing these activities until before COVID*?*) Yes*.*"* (man, 62 years old).

As in the previous process of living with RA, in which the multiple effects of the disease were implicated in the copying, participants also narrated different strategies to deal with the complex demands arising during the COVID-19 pandemic. They were aimed at emotional management, seeking information about the virus and disease, its health consequences and better prevention strategies, and protecting the families. Strategies of managing the SRH demands in the pandemic were mentioned less frequently.

“*(*. . .*) I learned a lot to manage my emotions*. *(*. . .*) I tried to control myself so as not to get sick because I said*: *The moment I get sick and end up in the emergency room*, *it is not going to be a very good option*, *for my husband who is far away*. . ., *for my mother*, *I am with her*, *well no*, *why distress them*, *right*? *(*. . .*) I looked for information*, *retook COVID courses (*. . .*)*.*"* (woman, 40 years old).

### SRH from an integral and narrative perspective of patients living with RA

**Fig 5:**
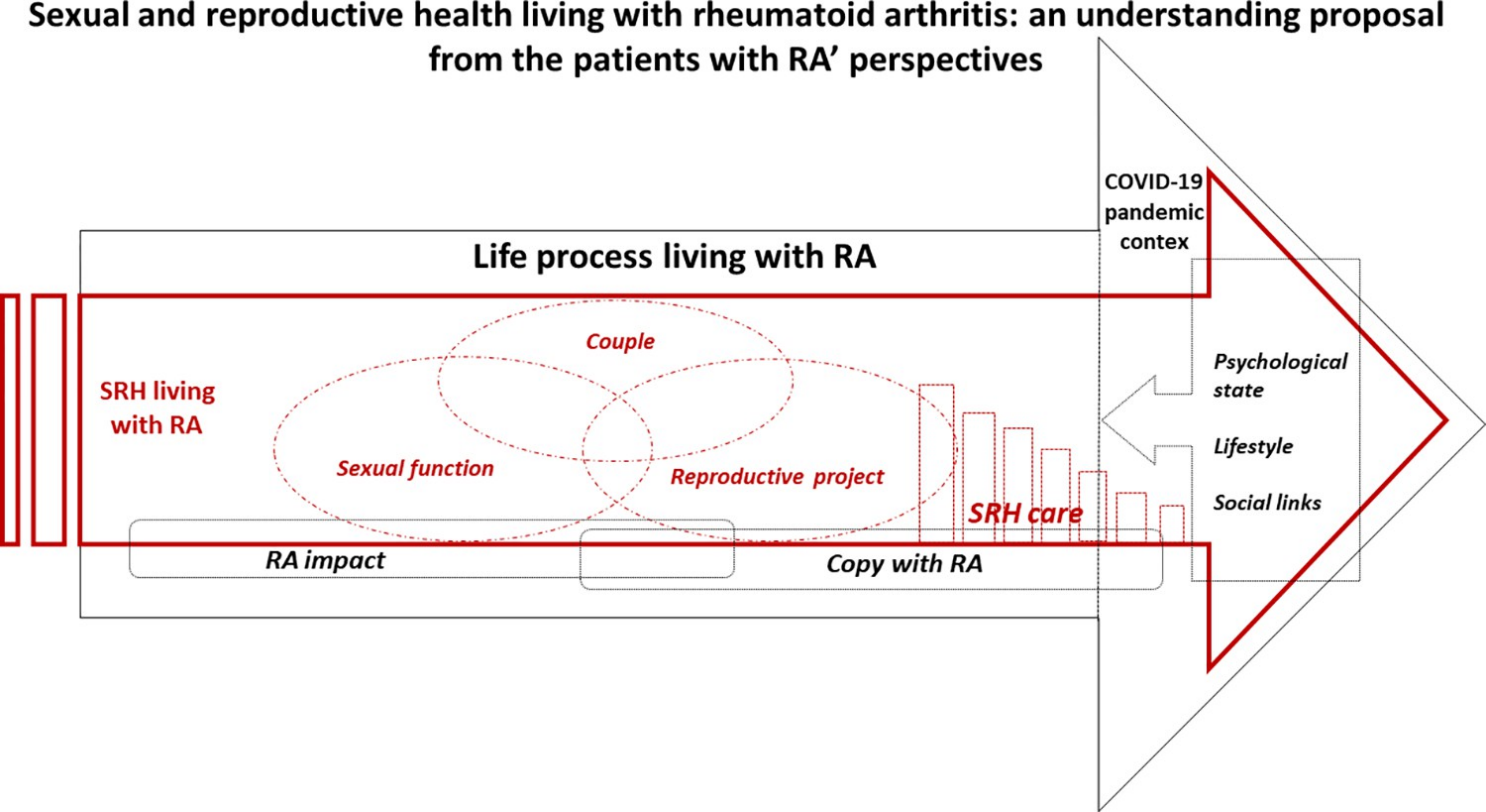
Sexual and reproductive health from an integral and evolutionary perspective of patients with RA.

In-depth interviews were conducted using a life history approach, having the SRH as the main driving line in the RA biography. From this framework, we proposed an understanding model based on interpreting the participants’ SRH narratives. In this model, their SRH experiences are embedded across their RA biography and integrated with the RA impact and the copy with the disease process. These intertwined experiences were also exposed during the COVID-19 pandemic when the participants’ biopsychosocial resources were challenged. The couple, the sexual function, and the reproductive project were the SRH fundamental areas from the patients’ perspectives, and the healthcare process was settled as a significant environment to enhance their SRH (**Fig 5**).

As a general finding, SRH contents emerged due to inquiring, not spontaneously. They were linked to some specific areas, such as the multi-layer RA impact, the strategies for coping with RA, and the disease healthcare before and during the COVID-19 pandemic. SRH contents did not emerge as part of the disease onset.

Patients structured the contents regarding RA’s impact on their SRH and coping with these demands in three intertwined areas: The dynamic of the couple, the sexual function, and the reproductive project. The first two areas were built as less implicated in the healthcare processes, showing two meanings: 1) as areas less related to health and 2) as poorly managed in the healthcare process. However, the reproductive project area was more linked to healthcare from the patient’s views and structured as insufficiently driven by the healthcare professionals.

From this framework of the SRH history in the process of living with RA, patients referred that the last stage during the COVID-19 pandemic added some SRH burdens due to its general burden expressed in its psychological, social, and lifestyle effects. These had specific SRH expressions (once again) on their couple dynamics, their sexual function, and their reproductive project, with the addition that they received less support from the healthcare services in general and specifically at the INCMyN-SZ. Some reasons expressed were healthcare interruption during the COVID-19 pandemic, increased health costs because of limited access to the Institution and the higher use of private services, a switch to telemedicine, and some patients losing their follow-up.

## Discussion

The current study was performed on Mexican outpatients with RA during the COVID-19 pandemic and explored their SRH biography while living with the disease from a narrative and integral approach. To our knowledge, no studies have comprehensively examined SRH in such a unique context among patients with rheumatic diseases, validating their perspectives and experiences and how they structure them. Research on SH continues to be scarce, especially among people chronically ill [26], for whom sexuality and intimacy remain of significant importance relatively because of their disruption by sexual dysfunctions, discomforts, and relationship issues [27].

The results of the present study point to some important considerations.

First, SRH was a relevant area throughout patients´ lives and was impacted by the disease, primarily with deleterious effects. Still, it emerged differently according to the RA life stage. At disease onset, when patients struggled with identifying and seeking first aid sources during the first disease-related manifestations, RA expression on SRH did not appear relevant enough to be recognized. The explicit inquiry during the in-depth interviews supported this argument. Nevertheless, once the healthcare process was adopted and a new stage of living with RA began (after diagnosis and treatment recommendations were shared with the patients), contents related to different sexual and reproductive areas were more frequently and deeply expressed. It is possible that the broad and profound impact generated by RA at disease onset (not limited to physical aspects) turned the SRH into a temporarily not prioritized theme. Here, it could be critical to consider some psychological processes related to the cognitive structuring of the RA symptoms, the loss and grief experiences, and the individuals’ priority structure as possible dimensions that put SRH issues on the sideline. Quality of life (QoL) is often identified as impaired in patients with RA, and this has been related to physical function, disease duration, and disease activity level [24,25]. Meanwhile, psychological comorbidities such as anxiety and depression also impact the QoL [28,29].

Current qualitative studies conducted with patients living with other chronic illnesses have also shown that they experience sexual adversities attributed to the physical, psychosocial, and direct sexual effects of the underlying chronic illness [30–34]. Nevertheless, while these studies have not focused on the process of living with the disease, the possibility of identifying the mechanisms by which they could impact SRH through different disease stages has been limited.

Second, it is interesting how some themes emerged as closely linked, such as the "RA onset: Absence of SRH contents" with "Healthcare for RA: Emerging SRH contents" and "RA’s impact: Proliferation of SRH contents" with "Coping with the process of living with RA: SRH-related strategies." This finding is congruent with the individual’s active position when facing life circumstances that exceed their habitual resources, expressed in using various strategies to cope with them. Participants tended to seek aid through different resources (including healthcare) for handling the RA’s physical and psychological expressions, including a system of biopsychosocial coping strategies incorporated into their daily lives. Several studies about the coping processes in patients with RA also showed the varied strategies used, including active, emotional, cognitive, and social resources [35–38]. This wide range of copy strategies has also been described in other qualitative studies with populations living with diverse chronic illnesses related to their SH issues [30–34]. Specifically, in the RA field, some authors have identified factors related to the use of specific coping strategies, such as gender, age, education, the duration of the disease, ailments experienced, knowledge of RA, and the attitude towards the disease [35,37], as well as its influence on psychological distress, mental and physical wellbeing, and the QoL [37,38].

Third, the sexual and reproductive effects of RA emerged intertwined with other physical, psychological, and social deleterious effects experienced in daily life. Also, they triggered a system of coping strategies to restore the lost balance. The construction of the main SRH areas impacted by RA (the sexual function, the couple dynamics, and the reproductive project) as private may have impeded spontaneous emergency of these contents during the interviews. This situation could be exacerbated in the healthcare environment because of the preponderant biomedical perspective, the traditional asymmetric doctor-patient relationship, and the reduced time for an integral approach to the patient. Although most of the study participants were living with another chronic condition, their primary SRH references were linked to RA, which might be explained by the interview focusing on RA attribution. In addition, considering the constellation of participants’ issues related to health, it may also be argued that RA exhibited more complex biopsychosocial challenges compared to other comorbidities.

Some qualitative studies have highlighted the lack of visibility and support for the sexual well-being of people living with cardiovascular diseases [30], the fact that SH is not addressed during healthcare in patients with osteoarthritis [31] and that breast cancer survivors do not receive any information from healthcare providers regarding sexual issues and complications caused by induced menopause, and how to deal with them [33]. These offer a clear indication that SH problems of people with chronic conditions are a public health concern and, therefore, require the proactive support of health professionals [26]. However, these studies have not identified differences in how SH and RH aspects are assisted.

The current study showed that reproductive aspects are more visualized and assisted during healthcare. The development of a consensus worldwide and the efforts to integrate them as part of healthcare proves this claim [39], even when the rates of reproductive health in patients with RA are far from acceptable [11]. On the other hand, several studies have shown how RA is associated with sexual disorders and the impairment of each sexual domain of the global sexual function [40,41]. However, they remain primarily underdiagnosed in daily clinical practice [40,42]. Patient´s identification of the healthcare environment as a valuable resource for dealing with RA challenges, including those related to the SRH, allows reinforcing actions aimed at exploring and driving these topics as part of integral healthcare from a human rights approach and validating the individual views and the specific needs at each disease stage.

Fourth, the COVID-19 pandemic appeared from the patients’ narratives as a global context that increased the burden of RA, which SRH impact was mediated by the pandemic deleterious effects on healthcare, RA-related vulnerability, lifestyle changes, and the resultant psycho-affective state and QoL impairment. This situation worsened because of the COVID-19 clinical care prioritization and everyday healthcare interruption, which limited the systematic assistance to patients with RA and other chronic comorbidities. The ORCID from the INCMyN-SZ, a national referral center for rheumatic diseases located in México City where more than 7,000 patients receive health care, interrupted the in-person healthcare and moved to phone medical consultations when the Mexican government declared the Institution a dedicated COVID-19 hospital. Since June 2020, the OCDIR has been reinstalled, and currently, most of the patients receive face-to-face consultations [43].

Patients with RA might be at higher risk of developing COVID‐19 and severe COVID‐19 [28], related to the overall impairment of the immune system combined with the iatrogenic effect generated by glucocorticoids and immunosuppressive drugs [44,45]. In addition, patients have been affected by changes in access to care, telemedicine, drug shortages, anxiety, and social isolation, which might have contributed to disease flares during the COVID-19 pandemic [46,47]. Several studies conducted in the general population showed higher rates of psychological distress, anxiety, and depression related to changes in everyday activities and lifestyle [48,49]. Studies aimed to assess psychological variables of patients with RA have also shown an increase in distress, anxiety, and depression compared to the pre-pandemic period, associated with biological use, higher physical disability, specific symptoms, comorbidities, and higher risk of infection perception, compared to the healthy population [50,51]. In agreement with our results, some of the documented strategies in RA patients to manage the challenges of the COVID-19 pandemic included taking measures to continue self-care activities while preventing infections, managing emergent emotions, and adapting to the new ways of receiving healthcare [52]. Few studies have explored variables of SRH in patients with RA. Investigations carried out during the COVID-19 pandemic have shown increased impaired sexual function related to age, disease duration, comorbidities, disability, higher levels of anxiety and depression, and reduced quality of life [53–56].

Similarly, studies in the general population have shown lower sexual function scores compared to the pre-pandemic period, decreased frequency of sexual intercourse [57], decreased sexual satisfaction, increased sexual difficulties, and sexual stress during lockdown [58]. Also, access to contraceptive methods became complex, and the negative sexual and reproductive health experiences increased during the pandemic [59]. Hence, it is expected that the physical, psychosocial, and sexual health indicators in patients with RA could be even more affected during the COVID-19 pandemic.

Finally, the proposal of an SRH understanding model based on the integrative interpretation of the experiences and perceptions of a group of patients with RA allows us to better comprehend this crucial health area from a process and dynamic perspective. It provides a valuable tool to build adjusted and contextual interventions. Some proposals have been focused independently on the social, psychological, and physical effects and/or coping strategies for RA, as well as on some psychosocial aspects involved in the process of living with the disease [60,61]. Other proposals integrate biomedical functions into psychological variables, such as explaining the adjustment to chronic illness [17] and intervening in disability [62]. To our knowledge, this is the only proposed model that focuses explicitly on SRH aspects in patients with RA based on their narratives through their illness biography.

In conclusion, the SRH is relevant for female and male patients with RA while living with the disease. However, it appeared with different priorities throughout their biography and was not recognized during RA’s first stages (onset). The sexual function, the couple’s relationship, and the reproductive health were the main SRH areas impaired by the disease from the outpatients’ perspectives, which burden increased during the COVID-19 pandemic. Healthcare was considered an essential but still deficient support resource for their SRH. Considering the results of the present study, it is crucial to recognize that not always, and in all moments, SRH is a priority for patients living with RA. However, in the same way, sustaining a holistic and systematic healthcare approach that validates the SRH as part of integral health is relevant. In this sense, giving assistance related to family planning for all patients at reproductive age, managing sexual discomforts and dysfunctions in sexually active patients, especially with couples, and providing integral and scientific-based sexual education from an ethical perspective for all patients must be core health care actions with patients with RA. The results also lead to recommend conforming multidisciplinary health teams to provide regular and integral health care for patients with RA. Specifically, to reinforce the health teams with different medical specialties (including those related to the SRH) and with professionals from the psychological and social area to sustain a patient-centered approach.

The present research has some limitations related to the study design, which does not allow generalization of the findings. Also, the study design from a narrative and evolutionary perspective favors memory and accessibility biases. However, the broad, deep, and sequential character of the proposed qualitative approach, designed and conducted rigorously, allowed the description and interpretation of the specific results to structure a comprehensive model of the topic and contributed to the knowledge of SRH RA patients.

## Supporting information

S1 Table Standards for Reporting Qualitative Research (SRQR). (PDF)
